# The Pathogenesis and Immune Evasive Mechanisms of Equine Herpesvirus Type 1

**DOI:** 10.3389/fmicb.2021.662686

**Published:** 2021-03-04

**Authors:** Kathlyn Laval, Katrien C. K. Poelaert, Jolien Van Cleemput, Jing Zhao, Annelies P. Vandekerckhove, Annick C. Gryspeerdt, Barbara Garré, Karen van der Meulen, Hossein B. Baghi, Haileleul N. Dubale, Ines Zarak, Eline Van Crombrugge, Hans J. Nauwynck

**Affiliations:** ^1^Department of Virology, Parasitology and Immunology, Faculty of Veterinary Medicine, Ghent University, Merelbeke, Belgium; ^2^Division of Virology, Department Infectious Diseases and Immunology, Faculty of Veterinary Medicine, Utrecht University, Utrecht, Netherlands; ^3^HIV Cure Research Center, Department of Internal Medicine and Pediatrics, Ghent University and Ghent University Hospital, Ghent, Belgium; ^4^Shenzhen International Institute for Biomedical Research, Shenzhen, China; ^5^DGZ Vlaanderen vzw, Torhout, Belgium; ^6^Equipe Veterinary Practice, Equi Focus Point Belgium, Vlamertinge, Belgium; ^7^Haras de la vie, Koewacht, Belgium; ^8^PERSEUS bvba, Sint Martens Latem, Belgium; ^9^Infectious and Tropical Diseases Research Center, Tabriz University of Medical Sciences, Tabriz, Iran; ^10^College of Veterinary Medicine and Agriculture, Addis Ababa University, Bishoftu, Ethiopia

**Keywords:** pathogenesis, immune evasion, prevention, therapies, Equine Herpesvirus type 1

## Abstract

Equine herpesvirus type 1 (EHV-1) is an alphaherpesvirus related to pseudorabies virus (PRV) and varicella-zoster virus (VZV). This virus is one of the major pathogens affecting horses worldwide. EHV-1 is responsible for respiratory disorders, abortion, neonatal foal death and equine herpes myeloencephalopathy (EHM). Over the last decade, EHV-1 has received growing attention due to the frequent outbreaks of abortions and/or EHM causing serious economical losses to the horse industry worldwide. To date, there are no effective antiviral drugs and current vaccines do not provide full protection against EHV-1-associated diseases. Therefore, there is an urgent need to gain a better understanding of the pathogenesis of EHV-1 in order to develop effective therapies. The main objective of this review is to provide state-of-the-art information on the pathogenesis of EHV-1. We also highlight recent findings on EHV-1 immune evasive strategies at the level of the upper respiratory tract, blood circulation and endothelium of target organs allowing the virus to disseminate undetected in the host. Finally, we discuss novel approaches for drug development based on our current knowledge of the pathogenesis of EHV-1.

## Equine Herpesvirus Type 1 (EHV-1)

In 1933, Dimock and Edwards documented epidemic virus abortion in mares in Kentucky (Dimock and Edwards, 1933). The virus was first designated as “equine abortion virus” and later renamed equine herpesvirus type 1. EHV-1 was first isolated in 1966 from cases of abortion and paralysis (Saxegaard, 1966). The virus was recognized by the Herpesvirus Study group of the International Committee for Taxonomy of Viruses in 1988 (Roizman et al., 1992). EHV-1 is among nine equid herpesviruses that have been identified so far (Davison et al., 2009). EHV-1 shares its classification with other animal herpesviruses of agricultural importance including bovine herpesvirus type 1 (BHV-1) and pseudorabies virus (PRV). The EHV-1 virion is 200–250 nm in diameter and consists of four main structural components characteristic of herpesviruses: genome, capsid, tegument, and envelop. The viral genome consists of a linear double stranded DNA of 150 kbp. EHV-1 virion structure, replication cycle and cell-associated spread are not covered in this review, as several excellent reviews on alphaherpesviruses already addressed these topics in more detail (Whitley and Roizman, 2001; Mettenleiter et al., 2009).

## The Pathogenesis of EHV-1

Here, we provide an overview of the key steps in the pathogenesis of EHV-1 in the horse that will be relevant to thoroughly understand its immune evasive mechanisms.

### Introduction

EHV-1 is a highly contagious pathogen and is usually transmitted via direct contact with infectious secretions (saliva, nasal discharge) or via inhalation of infectious aerosols (Patel et al., 1982). Fetal or placental tissues that contain high virus loads may also serve as a possible source of infection (Reed and Toribio, 2004).

### EHV-1 Primary Replication in the Upper Respiratory Tract (URT)

Upon entry into the host, EHV-1 first replicates in a restricted plaquewise manner in the epithelial cells lining the URT, including nasal septum, nasopharynx, and trachea (van Maanen, 2002; Gryspeerdt et al., 2010; Figure 1.1a). *In vivo*, EHV-1-induced plaques were observed in the epithelium of the nasal mucosa starting from 2 to 7 days post-inoculation (dpi) (Gryspeerdt et al., 2010). In *ex vivo* experiments, single infected epithelial cells were visible at 12 h post-inoculation (hpi) and EHV-1-induced plaques were observed in the epithelium of equine nasal and nasopharyngeal explants starting from 24 hpi (Vandekerckhove et al., 2010; Negussie et al., 2016). Primary EHV-1 infection of several tissues of the URT results in the destruction and erosion of the epithelium and in nasal shedding starting from 1 to 14 dpi (Gibson et al., 1992; Gryspeerdt et al., 2010; Figure 1.1b). Ultimately, the destruction of the respiratory epithelial cells and local inflammation causes mild clinical symptoms (e.g., serous nasal discharge and fever) in the horse after 2–10 dpi (Allen and Bryans, 1986). Most EHV-1 infections are self-limiting in adult horses and accompanying respiratory symptoms disappear from 9 to 12 dpi (Gibson et al., 1992). However, young horses can develop more severe clinical symptoms consisting of high fever, swelling of the submandibular and retropharyngeal lymph nodes (Coggins, 1979; Patel et al., 1982). The nasal discharge can become mucopurulent, due to secondary bacterial infections, and contribute to the development of rhinopneumonitis (Thomson et al., 1979; McGavin and Zachary, 2007).

**FIGURE 1:**
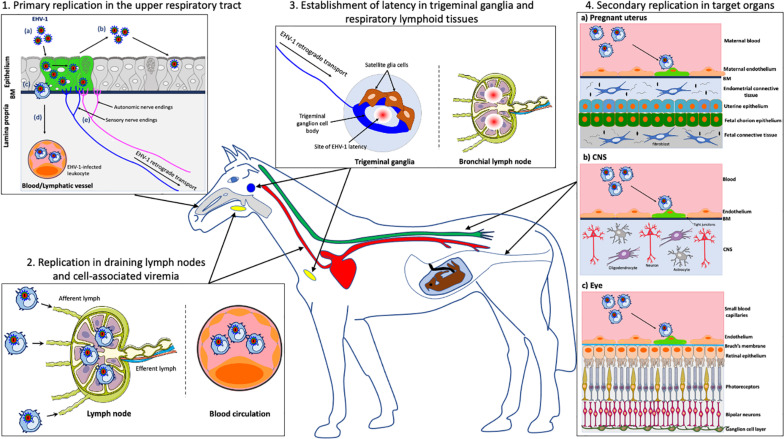
Schematic representation of the pathogenesis of EHV-1 in the horse. **(1)** Primary EHV-1 replication in the epithelial cells of the upper respiratory tract: (a) EHV-1 infection (green); (b) Viral spread within the respiratory epithelium and viral shedding; (c) EHV-1 crosses the basement membrane (BM) and penetrates the lamina propria via the use of single infected leukocytes; (d) EHV-1 reaches the blood circulation and draining lymph nodes; (e) EHV-1 enters nerve endings of the peripheral nervous system and spreads in the retrograde direction to the trigeminal ganglia (TG). **(2)** EHV-1 replication in the draining lymph nodes and establishment of a cell-associated viremia in peripheral blood mononuclear cells (PBMC). **(3)** Establishment of EHV-1 latency in TG neurons and respiratory lymphoid tissues. **(4)** Via a cell-associated viremia in PBMC, EHV-1 is transported to target organs such as the pregnant uterus (4a), the central nervous system (CNS) (4b) or the eye (4c), where it initiates a secondary replication in the endothelial cells lining the blood vessels of this organ. *gray*, *respiratory tract; red*, *blood circulation; yellow*, *lymph nodes; blue*, *TG; green*, *spinal cord.* Drawings are partially based on SMART servier medical art templates.

Recently, Van Cleemput and colleagues demonstrated that certain environmental circumstances stimulate the replication of EHV-1 in the URT. They showed that plant pollen contain proteases that selectively and irreversibly alter intercellular junctions (ICJ) of columnar equine respiratory epithelial cells (Van Cleemput et al., 2019a). The authors demonstrated that EHV-1 infection of *ex vivo* respiratory mucosal explants is greatly enhanced following pollen protease pretreatment, suggesting that pollen-induced loss of respiratory epithelial barrier function facilitates EHV-1 invasion. Interestingly, pollen concentrations in the outdoor air have been shown to peak between late winter and spring, a period that coincides with high incidence of seasonally observed EHV-1-associated symptoms (e.g., respiratory disease, abortion and EHM) (Goehring et al., 2006; D’Amato et al., 2007). Similarly, it was demonstrated that pretreatment of *ex vivo* respiratory mucosal explants and EREC with deoxynivalenol (DON), a mycotoxin mainly present in equine feeds, alters respiratory epithelial integrity and predisposes these cells to EHV-1 infection (Van Cleemput et al., 2019b). It was proposed that the inhalation of mycotoxins from contaminated hay, grains and roughage disrupts the respiratory epithelial integrity and subsequently promotes EHV-1 infection. Most importantly, the authors dissected the mechanisms of EHV-1 invasion through the respiratory epithelium. Equine mucosal explants were first treated with EGTA or N-acetylcysteine (NAC), two chelating agents, to disrupt respiratory epithelial ICJ and were subsequently inoculated with EHV-1. The number of EHV-1 plaques, the plaque latitude and infectious virus titer were significantly increased in explants pretreated with EGTA or NAC compared to untreated ones (Van Cleemput et al., 2017). Interestingly, the authors demonstrated that disruption of ICJ results in enhanced EHV-1 binding to explant basolateral surfaces. Consequently, it was hypothesized that EHV-1 binding/entry receptor is expressed basolaterally on respiratory epithelial cells and is only exposed when the integrity of the epithelium is compromised. So far, the EHV-1 basolateral receptor remains uncharacterized.

### EHV-1 Replication in Draining Lymph Nodes and Cell-Associated Viremia

Following infection of the respiratory epithelium, EHV-1 crosses the basement membrane (BM) by the use of single infected leukocytes (Kydd et al., 1994; Vandekerckhove et al., 2010; Figure 1.1c). These cells were mainly identified as CD172a^+^ cells (myeloid origin) followed by T lymphocytes and B lymphocytes (Gryspeerdt et al., 2010; Vandekerckhove et al., 2010; Baghi et al., 2014). Upon crossing the BM, EHV-1-infected leukocytes penetrate the connective tissues and reaches the bloodstream and the draining lymph nodes (Figure 1.1d). Within 24–48 hpi, EHV-1 antigens as well as infectious virus can be detected in submandibular, retropharyngeal and bronchial lymph nodes. The infection is amplified in the draining lymph nodes with discharge of infected leukocytes, via the efferent lymph, into the blood circulation (Kydd et al., 1994). As a result, EHV-1 initiates a cell-associated viremia in peripheral blood mononuclear cells (PBMC) to disseminate in the host (Siedek et al., 1999; van der Meulen et al., 2000; Figure 1.2). Viremia can be detected starting from 1 dpi and persists for 14 days (Gryspeerdt et al., 2010). The cell-associated viremia is a prerequisite for EHV-1 spread to target organs such as the central nervous system (CNS) and/or pregnant uterus. The spread of EHV-1 to the local lymph nodes and in the bloodstream leads to clinical symptoms including lymph node swelling and fever (Patel et al., 1982; Allen and Bryans, 1986).

### Establishment of a Latent EHV-1 Infection in the Peripheral Nervous System (PNS) and in Respiratory Associated Lymphoid Tissues

After primary replication in the horse respiratory epithelium, EHV-1 enters nerve endings of the PNS, including those coming from the trigeminal ganglia (TG), sympathetic and parasympathetic neurons that innervate the epithelium (Figure 1.1e; Van Cleemput et al., 2017). EHV-1 particles travel via retrograde transport to the sensory and autonomic peripheral ganglia. A hallmark of herpesviruses is the establishment of a reactivable, latent infection in its host (Grinde, 2013). Therefore, EHV-1 establishes a lifelong latent infection in horse PNS neurons (Figure 1.3). An *in vivo* study of Slater and colleagues presented evidence of a latent EHV-1 infection in horses. Two months following the initial intranasal EHV-1 inoculation, the authors administered a reactivation stimulus (e.g., dexamethasone) to asymptomatic ponies. EHV-1 infectious virus and viral DNA were subsequently detected in the TG of infected ponies by cocultivation and PCR assays (Slater et al., 1994a,b). These results were confirmed by another study demonstrating the presence of specific latency-associated transcripts (LAT) in TG of EHV-1-infected horses by *in situ* hybridization (Baxi et al., 1995). EHV-1 has also been shown to establish latency in respiratory associated lymphoid tissues (e.g., mandibular, retropharyngeal and bronchial lymph nodes). Latent EHV-1 was detected by PCR and recovered by co-cultivation from lymphoid tissues draining the respiratory tract of experimentally inoculated ponies (Welch et al., 1992; Kydd et al., 1994; Slater et al., 1994a). Additional studies stating that EHV-1 establishes latency in lymphoid tissues, failed to conclusively demonstrate the presence of LATs in these tissues (Pusterla et al., 2010, 2012; Goehring, 2017; Giessler et al., 2020). Finally, circulating T lymphocytes have been defined as a predominant site of EHV-1 latency. Using southern hybridization and PCR techniques, Chesters and colleagues first demonstrated that PBMC express a putative LAT antisense to and overlapping the 3′ end of the EHV-1 immediate-early gene (ORF64) (Chesters et al., 1997). An *in vitro* study showed that EHV-1 reactivates from CD8^+^ T lymphocytes upon mitogen stimulation (e.g., phytohaemagglutin ad pokeweed mitogen) (Smith et al., 1998). It was estimated that 1/50,000 PBMC are latently infected with EHV-1. However, these results must be interpreted with caution as the authors also failed to demonstrate the presence of LAT in these cells and/or confirm the findings obtained by Chesters and colleagues.

Upon stress-induced reactivation months or years after primary infection, EHV-1 replication occurs and particles spread back from infected T lymphocytes or from infected TG neurons through anterograde transport to the respiratory epithelium where the infection is initiated. The presence of an EHV-1 receptor located at the basolateral side of the respiratory epithelium is a key advantage for the virus to efficiently bind and infect epithelial cells upon reactivation (Van Cleemput et al., 2017). So far, there is no direct evidence of EHV-1 neuronal spread from the PNS (e.g., TG) to the CNS (e.g., brain). Cycles of EHV-1 latency and reactivation in horses lead to the shedding of infectious virus and transmission to new hosts, allowing the virus to be maintained in herds. In addition, latently infected cells are masked from immune surveillance and constitute a permanent reservoir of the virus that is difficult to eliminate by conventional antiviral therapies and vaccine strategies. It is now estimated that the majority of horses (> 60%) are latently infected by EHV-1 (Lunn et al., 2009).

### Secondary EHV-1 Replication in the Pregnant Uterus, CNS and/or Eye

Once in the blood circulation, infected leukocytes can adhere and subsequently transfer EHV-1 to the endothelial cells (EC) lining the blood vessels of target organs such as the pregnant uterus or CNS. The infection of EC located in the vasculature of the late-gravid uterus or CNS is mediated by cell-to-cell contacts between infected PBMC and EC (Goehring et al., 2011). The adhesion molecules present on the surface of both EC [e.g., intracellular adhesion molecule (ICAM), E-selectin, P-selectin] and leukocyte [e.g., α_4_β_1_ (VLA-4), α_L_β_2_ (LFA-1), and α_v_β_3_ integrins] have been shown to play an important role in the infection of the vascular endothelium (Smith et al., 2001; Laval et al., 2015b).

Secondary replication in the EC of the pregnant uterus causes vasculitis and multifocal thrombosis that particularly affect small arteriolar branches in the glandular layer of the endometrium at the base of the microcotyledons (Edington et al., 1991; Smith et al., 1992, 1993; Figure 1.4a). This event leads to avascular necrosis and edema of the endometrium. A widespread EC infection can cause detachment of the fetal membranes, thus leading to the abortion of a virus-negative fetus. Less extensive uterine vascular pathology allows EHV-1 to invade the fetus through the uteroplacental barrier and leads to the abortion of a virus-infected fetus. In EHV-1-positive fetuses, infected EC are present in blood vessels of the allantochorion and umbilical cord (Smith et al., 1997). The aborted fetus shows multiple lesions, including subcutaneous edema, pulmonary edema and splenic enlargement (Corner et al., 1963; Machida et al., 1997). At late stage of gestation, transplacental EHV-1 infection often results in the delivery of a live infected foal that dies within a few days. Whether or not abortion occurs may depend on the hormonal activity during pregnancy. The production of high levels of cortisone, progesterone and estrogens at late stage of pregnancy may alter the immune system of the mare (Smith et al., 1996). EHV-1-induced abortion typically occurs in the last trimester of pregnancy. The incubation period of abortion following viremia, arising from either a primary infection or a reactivation of latent virus, varies between 9 days and 4 months (Allen, 1999, 2002). EHV-1-induced abortions usually occur in single mares within a group, suggesting that abortion resulted from viral reactivation rather than from newly acquired respiratory infection (Doll and Bryans, 1963). Mares may experience respiratory symptoms and fever prior to abortion but can also abort without showing any clinical symptoms. The reproductive potential of the mare is usually not affected, and the virus is rapidly cleared from the reproductive tract.

Secondary replication in the EC lining the blood vessels of the CNS can cause vasculitis with or without local hemorrhage and thrombo-ischemic necrosis, in the brain and spinal cord (Figure 1.4b; Edington et al., 1986; Wilson, 1997). The lack of nutrients and oxygen and the elevated levels of inflammatory cytokines cause neurons to degenerate, eventually leading to equine herpes myeloencephalopathy (EHM) (Jackson and Kendrick, 1971; Wilson, 1997). Neurological symptoms go from ataxia to a complete fore and hind limb paralysis. Other clinical signs comprise fecal and/or urinary incontinence, head tilting, tail paralysis, distal limb edema and blindness (Jackson and Kendrick, 1971; van Maanen et al., 2001; Borchers et al., 2006; Gryspeerdt et al., 2011). The prognosis for non-recumbent horses is favorable but full recovery can take months. However, recumbent horses usually develop fatal complications and require euthanasia. The incubation period of EHV-1-induced neurological disorders can vary between 6 and 8 days (Mumford et al., 1994). Several risks factors, including the age (elder), breed (Standardbred), gender (female), season (late autumn, winter, spring), and type of EHV-1 strain (neurovirulent) have been associated with an increased risk to develop EHM (Goehring et al., 2006; Henninger et al., 2007; Allen, 2008; Perkins et al., 2009; Pusterla et al., 2016). The neurovirulent EHV-1 strain presents a single nucleotide polymorphism (SNP) in the catalytic subunit of the viral DNA polymerase (ORF30), causing a substitution of asparagine (N) by aspartic acid (D) at amino acid position 752 (Nugent et al., 2006). It was demonstrated that more than 86% of neuropathogenic outbreaks are caused by strains encoding D752 and more than 95% of non-neuropathogenic outbreaks were caused by virus strains encoding DNA pol N752. Horses infected with neurovirulent strains also showed a higher and longer viremia compared to those infected with non-neurovirulent strains and were more likely to develop neurological disorders (Allen and Breathnach, 2006). These results were confirmed by targeted mutation of the D_752_ to the N_752_ genotype in a neurovirulent isolate which resulted in attenuation of virulence, reduced levels of viremia and reduced capacity to cause EHM (Goodman et al., 2007).

Finally, secondary replication of EHV-1 in the vasculature of the eye can cause multifocal chorioretinal lesions in infected horses (Figure 1.4c; Matthews, 2004). This type of lesion is typically caused by endothelial damage with subsequent ischemic injury to the chorioretina that may result from direct infection of the vascular endothelium following viremia. Most EHV-1-induced ocular infections are subclinical but sometimes, diffuse lesions may lead to extensive retinal destruction and blindness. The frequency of EHV-1 ocular lesions varies between 50 and 90% in experimentally infected horses (Hussey et al., 2013).

## The Mechanisms of EHV-1 Immune Evasion

In this section, we discuss the current knowledge on the pathogenesis of EHV-1 with an emphasis on its immune evasive strategies developed at the level of the URT, blood circulation and endothelium of target organs in order to survive and disseminate undetected in the host.

### Restricted EHV-1 Replication in the URT: A Strategy to Control Innate Immune Defenses

Recently, Van Cleemput and colleagues showed that EHV-1 exploits antimicrobial equine β-defensins (eBD) to initiate viral replication and spread within the URT. Pretreatment of primary respiratory epithelial cells (EREC) with eBD increased EHV-1 virion binding and infection of these cells. It was demonstrated that EHV-1 virions are resistant to the antimicrobial properties of eBD through the action of EHV-1 gM, which stabilizes the viral envelope and protects it from eBD permeabilization (Van Cleemput et al., 2020). While EHV-1 can infect the respiratory epithelium, previous *in vivo*, *ex vivo* and *in vitro* studies showed that its replication is somehow restricted (Gryspeerdt et al., 2010; Vandekerckhove et al., 2010; Hussey et al., 2014). The limited replication of EHV-1 represents a finely tuned viral strategy to control the innate immune responses at the primary site of infection (Figure 2.1). On the one hand, this strategy allows a limited number of EHV-1 particles present in the epithelium to reach the cell bodies of TG neurons and to efficiently establish latency. The virus is also able to modulate the recruitment of circulating leukocytes to the URT. On the other hand, this strategy prevents the onset of a strong immune response that could be detrimental for the host. The interferon system (IFN) is the most effective mechanism of the innate response against viral infections, including EHV-1. Equine type 1 interferon (IFNα/β) has been detected in nasal secretions and serum of experimentally EHV-1-infected ponies the first 2 weeks post-inoculation (Edington et al., 1989; Chong and Duffus, 1992). The production of type I IFN was directly associated with a self-limited EHV-1 infection of the respiratory epithelium and short-duration of nasal shedding (Gryspeerdt et al., 2010). EHV-1 activated the IFN response in the URT by upregulating the expression of IFN-α mRNA and protein levels in EREC and respiratory mucosal explants (Hussey et al., 2014; Poelaert et al., 2018). However, it was demonstrated that EHV-1 modulates the IFN antiviral response in a virus strain-specific manner. The replication of EHV-1 neurovirulent strains was more efficient in explants treated with Ruxolitinib, an IFN signaling inhibitor, compared to non-treated explants. In contrast, the replication of EHV-1 non-neurovirulent strains was not altered in treated explants with plaque size similar to control group. The authors suggested that EHV-1 non-neurovirulent strains have developed an anti-IFN mechanism to focus on a more efficient replication at the level of the URT than neurovirulent strains.

**FIGURE 2:**
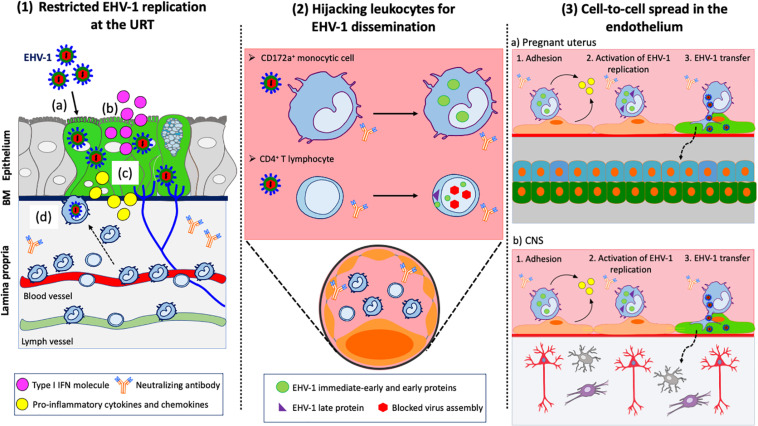
EHV-1 immune evasive strategies. **(1)** Restricted replication at the URT: (a) EHV-1 infects respiratory epithelial cells; (b) Infected epithelial cells produce type I IFN to limit viral spread within the upper respiratory tract (URT); (c) Infected epithelial cells produce pro-inflammatory cytokines and chemokines to recruit immune cells to the site of infection; (d) CD172a^+^ cells and T lymphocytes migrate to the URT. EHV-1 uses these cells to cross the basement membrane (BM) and penetrate the lamina propria. **(2)** Hijacking leukocytes for viral dissemination. EHV-1 silences its replication in immune cells to avoid detection by neutralizing antibodies in the blood circulation and draining lymph nodes. EHV-1 only expresses immediate-early and early proteins in CD172a^+^ cells. All classes of EHV-1 proteins are expressed in T lymphocyte but viral assembly is blocked, and no progeny virions are released. **(3)** Cell-to-cell spread in the endothelium of target organs: EHV-1 infected immune cells are transported via the blood circulation to the pregnant uterus (a) and CNS (b). The adhesion of infected immune cells to the endothelial cells lining the blood vessels of target organs activates viral replication. New EHV-1 progeny virions are released from infected immune cells and transferred to endothelial cells via (micro)fusion events. Dashed arrows represent further spread of virus particles through connective tissues or CNS. Drawings are partially based on SMART servier medical art templates.

Several *ex vivo* and *in vitro* studies further characterized the innate immune responses to EHV-1 infection in the respiratory epithelium. Soboll-Hussey and colleagues showed that EHV-1 induces the upregulation of toll-like receptors (TLR)-3 and TLR-9 as well as inflammatory cytokines (e.g., IL-1, TNF-alpha, and IL-6) and chemokines (e.g., IL-8, MCP-1) in EREC (Hussey et al., 2014). In nasal mucosal explants, EHV-1 elicited the production of chemokines (e.g., CCL2 and CCL5), already at 24 hpi, in order to attract target CD172a^+^ cells (Gryspeerdt et al., 2010; Zhao et al., 2017). Using a chemotaxis assay, it was demonstrated that EHV-1 upregulates the expression of CXCL9, CXCL10, eBD2, and 3 in EREC and induces the recruitment of CD172a^+^ cells and T lymphocytes to the site of infection (Poelaert et al., 2019c; Van Cleemput et al., 2020). Both studies indicated that primary replication of EHV-1 neurovirulent strains in the URT attracted significantly more CD172a^+^ cells and T lymphocytes to the infected epithelium than non-neurovirulent strains. These results are in accordance with a previous *ex vivo* study of Vandekerckhove and colleagues (Vandekerckhove et al., 2010). EHV-1 neurovirulent strains are likely to favor rapid infection of immune cells rather than efficient spread through the epithelium. So far, two EHV-1 glycoproteins (gG and gp2) have been shown to control the recruitment of leukocytes to the horse’s respiratory tract. EHV-1 gG is a chemokine binding protein (vCKBP), which binds to a broad range of chemokines, such as IL-8 and CXCL1, with high affinity and subsequently blocks their activity (Bryant et al., 2003). *In vivo*, an EHV-1 gG deleted mutant induces a more pronounced inflammatory response compared to the wild-type virus (von Einem et al., 2007). EHV-1 gG is also able to inhibit migration of equine neutrophils in response to equine recombinant IL-8 (Van de Walle et al., 2007). Using a functional cell migration assay, the infection of EREC with a EHV-1 deleted gG mutant was shown to increase the migration of monocytic cells and T lymphocytes to the infected site (Poelaert et al., 2019c). In addition, EHV-1 gp2 was identified as another viral protein with similar chemokine-binding activities as gG, that impedes the migration of immune cells to the URT. Besides, EHV-1 early protein pUL56 (encoded by EHV-1 ORF1) has also been shown to modulate the cytokine responses both *in vivo* and *in vitro* (Soboll Hussey et al., 2011; Hussey et al., 2014). pUL56 plays a major role in the downregulation of cell surface Major Histocompatibility Complex type I (MHC I) in infected cells (Ma et al., 2012; Huang et al., 2014, 2015). Soboll-Hussey and colleagues demonstrated that EHV-1 infection significantly down-regulates MHC I expression in EREC. Infection with a deleted ORF1 mutant partially restored MHC I expression and significantly increased chemokine expression and chemotaxis of immune cells to EREC (Soboll Hussey et al., 2014). Therefore, it was proposed that EHV-1 pUL56 has a dual function during infection. On one hand, this protein modulates the recruitment of leukocytes at the primary site of infection. On the other hand, it downregulates MHC I expression on infected cells and allows the virus to evade CTL-mediated cell lysis. This may explain why the virus can still cause a viremia in the presence of CTL precursors in the host (O’Neill et al., 1999; Kydd et al., 2006).

### Hijacking Leukocytes: The “Trojan Horse” Mechanism of EHV-1 Dissemination

Previous *in vivo* and *ex vivo* studies of the pathogenesis of EHV-1 demonstrated that the virus misuses single leukocytes from the URT to breach the BM into the connective tissues (Gryspeerdt et al., 2010; Vandekerckhove et al., 2010; Figure 2.2). It was demonstrated that the expression of late gC and gD proteins is hampered in these cells at early stages of infection (Gryspeerdt et al., 2012). These findings are in accordance with a study from van der Meulen and colleagues, showing that the expression of EHV-1 late proteins is hampered in circulating leukocytes (van der Meulen et al., 2006). These studies suggest that an early block in the replication cycle of EHV-1 protects infected leukocytes from efficient recognition by the immune system. This “Trojan horse” mechanism allows infected leukocytes to disseminate in the blood circulation despite the presence of virus-neutralizing antibodies. Still, the minority of PBMC expressing viral glycoproteins at their cell surface were found to be resistant to antibodies, suggesting that EHV-1 uses other strategies to evade antibody-dependent cell lysis (van der Meulen et al., 2003).

Laval and colleagues further investigated the mechanisms of how EHV-1 hijacks and modulates its replication in the main carrier cells: CD172a^+^ cells. The authors demonstrated that the replication of EHV-1 is highly restricted in these cells with less than 10% infected compared to fully susceptible rabbit kidney (RK-13) cells (Laval et al., 2015a, 2017). In addition, EHV-1 replication is mainly non-productive in these cells. EHV-1 non-neurovirulent strains, but not neurovirulent strains, delay their replication in CD172a^+^ cells at a very early time of infection. The gene expression of EHV-1 non-neurovirulent strains is silenced and tightly regulated by histone deacetylases (HDAC) in these cells. Chromatin remodeling by HDAC plays a key role in the regulation of viral gene expression during herpesvirus infections (Sarkar et al., 2006; Guise et al., 2013). These findings suggest that EHV-1 non-neurovirulent and neurovirulent strains use distinct immune evasive strategies in target cells. Silencing gene expression by HDAC might be an epigenetic strategy for EHV-1 non-neurovirulent strains to slow down their replication and persist longer in CD172a^+^ cells than neurovirulent strains. It was proposed that mutations in the coding sequences of some regulatory proteins of EHV-1 non-neurovirulent strains might alter their capacity to bind and subsequently phosphorylate HDAC and/or HDAC-containing complexes at viral promoters in CD172a^+^ cells. This may result in increased levels of HDAC and repression of EHV-1 gene expression, and thus may indirectly delay viral replication in these cells. However, the fact that both viral strains can still cause a viremia in vaccinated horses indicates that manipulation of HDAC is only one part of EHV-1’s strategy to bypass detection by the immune system. Laval and colleagues further investigated the molecular mechanisms underlying the restricted infection of EHV-1 in CD172a^+^ cells (Laval et al., 2016). Using dioctadecyloxacarbocyanine perchlorate (dio)-labeled EHV-1 particles, the authors characterized the entry mechanisms of EHV-1 in these cells. EHV-1 bound to a limited number of CD172a^+^ cells compared to RK-13 cells, suggesting that a block at the virus binding level might be partially responsible for the restricted viral replication in these cells. These results also suggest the presence of specific receptor(s) at the cell surface. Previous studies have already shown that EHV-1 uses a cell surface receptor that is distinct from any of the known alphaherpesvirus entry receptors (Frampton et al., 2005). MHC I has been identified as a functional entry receptor for gD in equine dermal and brain microvascular EC (Kurtz et al., 2010; Sasaki et al., 2011). However, a study from Azab and colleagues demonstrated that MHC I antibodies do not efficiently block EHV-1 entry into PBMC, indicating that different receptors are present on equine leukocytes (Azab and Osterrieder, 2012). Enzymatic removal of sialic acids present on the cell surface, but not heparan sulfate, inhibited EHV-1 infection of CD172a^+^ cells by 90–100%. It was proposed that EHV-1 interacts with a sialic acid-containing cell surface receptor expressed on these cells. Future work is still needed to determine which sialylated glycans are expressed on CD172a^+^ cells and involved in EHV-1 binding step. Specific EHV-1 glycoprotein(s) that serve as viral glycan-binding proteins should also be identified. EHV-1 gC has hemagglutination activity against equine red blood cells and is considered a potential viral lectin candidate for the binding of sialic acid residues on CD172a^+^ cells (Andoh et al., 2015). Moreover, the blockage of α_v_β_3_ integrin by neutralizing antibodies significantly reduced EHV-1 entry at a post-binding step. α_v_β_3_ integrin acts as a co-receptor for HSV1 and HCMV (Wang et al., 2005; Gianni and Campadelli-Fiume, 2012). The interaction between α_v_β_3_ integrin present on PBMC and the arginine, serine and aspartic acid (RSD) motif present in EHV-1 gD is known to trigger EHV-1 entry via endocytosis (Van de Walle et al., 2008). Therefore, it was proposed that EHV-1 uses α_v_β_3_ integrin to route its entry via endocytosis into CD172a^+^ cells in order to shorten its exposure at the cell surface. Finally, the authors demonstrated that EHV-1 enters CD172a^+^ cells via an endocytic mechanism that requires cholesterol, tyrosine kinase activity, actin polymerization, dynamin activity, and endosomal acidification, pointing toward a phagocytic mechanism. Phagocytic mechanisms have already been described for the entry of HSV1 and CMV into cells (Clement et al., 2006; Tiwari and Shukla, 2012). The formation of protrusions at the surface of CD172a^+^ cells mediated by actin polymerization may facilitate uptake of EHV-1 into the cell and protect the virus from neutralizing antibodies.

While CD172a^+^ cells are considered the main “Trojan horse” in the pathogenesis of EHV-1, T lymphocytes also serve as an important vehicle for viral dissemination. EHV-1 predominantly infects T lymphocytes (CD4^+^) with EHV-1 non-neurovirulent strains infecting twice as much cells (~10%) than neurovirulent strains (Poelaert et al., 2019b). Both type of strains efficiently replicated in T lymphocytes with the expression of all classes of viral proteins. However, viral glycoproteins clustered at the plasma membrane of T lymphocytes and formed aggregates. Viral capsids were also found to accumulate in the cell nucleus and the release of progeny virions was hampered. The authors proposed that the aggregation of membrane-bound viral proteins at the cell surface and the blockage of virus assembly represent two new immune evasive mechanisms that EHV-1 uses to prevent efficient recognition of infected cells by antibodies. Interestingly, contact of the infected T lymphocytes with either another T lymphocyte or CD172a^+^ cell activated viral assembly and egress. The transfer of infectious virus from T lymphocyte to target cell was mediated by the polarization of microtubule-organizing center (MTOC) and the accumulation of lymphocyte function-associated antigen 1 (LFA1) at the site of contact. These events resulted in the formation of a virological synapse between the two cells. The authors further demonstrated that EHV-1 hijacks the dynein motor proteins to catalyze the transport of viral progeny along the microtubule network that connects the cellular nucleus to the virological synapse. These findings show that cell-to-cell spread is an essential viral mechanism to disseminate within the host and evade immunosurveillance.

### EHV-1 Transfer From Leukocytes to Endothelial Cells: Unloading the Trojan Horse

EHV-1-induced abortion and neurological disease are direct results of viral spread from infected leukocytes to EC lining the blood vessels of target organs, and their subsequent infection. EHV-1 also relies on cell-to-cell spread to bypass antibody-mediated immune responses for efficient transmission to the endothelium (Figure 2.3).

Recently, several studies investigated the molecular mechanisms of EHV-1 transmission from leukocytes to EC. Laval and colleagues showed that EHV-1 infection significantly increases adhesion of CD172a^+^ cells to equine EC. Antibody-blocking experiments indicated that α_V_β_3_, α_4_β_1_, and α_L_β_2_ integrins partially mediate adhesion of infected CD172a^+^ cells to EC (Laval et al., 2015b). The authors proposed that the interaction of EHV-1 gD and α_V_β_3_ integrin expressed at the surface of CD172a^+^ cells activates PI(3)K and ERK/MAPK signaling pathways. This event mediates EHV-1 entry into CD172a^+^ cells at early time of infection. Later, activated integrins expressed on infected CD172a^+^ cells interact with EC ligands, facilitating immune cell adhesion to the endothelium. The PI3K signaling pathway plays an important role in the inflammatory response. PI3K isoform γ is highly expressed in leukocytes and triggers chemokine-mediated recruitment and activation of innate immune cells at inflammation sites (Hawkins and Stephens, 2015). It was therefore suggested that EHV-1 infection switches CD172a^+^ cells to a pro-inflammatory phenotype to promote their differentiation into macrophages, migration, and subsequent adhesion. However, another study demonstrated that the release of cytokines and chemokines is significantly reduced in PBMC upon EHV-1 infection (Pavulraj et al., 2020). Besides, EHV-1 induces procoagulant activity in equine monocytic cells (Yeo et al., 2013). Tissue factor (TF) is the primary activator of coagulation and is mainly produced by monocytes in an inflammatory state (Østerud, 2010). It was proposed that increased monocyte TF expression promotes their adhesion to the endothelium and this process may be involved in EHV-1-associated thrombosis. The adhesion of EHV-1-infected CD172a^+^ cells to EC was found to correlate with the production of pro-inflammatory cytokines, such as TNF-α, in the microenvironment (Laval et al., 2015b). The adhesion process is likely to create an inflammatory environment that facilitates the recruitment of additional monocytic cells to the endothelium. This inflammatory cascade may promote EC infection. Additional factors present in the local environment of the endothelium may influence the adhesion of leukocytes and transfer of virus to EC. It was demonstrated that cytokines (e.g., IL-2) and hormones (e.g., 17-estradiol and equine chrorionic gonadotropin) upregulate the expression of adhesion molecules on EC (Smith et al., 2002). Consequently, the hormonal activity and immune status of the pregnant mare may impact on the efficient infection of the endothelium and the development of abortion at late stage of pregnancy. Short-chain fatty acids (SCFA), such as sodium butyrate and sodium propionate, are metabolic end-products of the fermentation of dietary fibers. Recently, SCFA were found to downregulate the expression of ICAM-1 and VCAM-1 adhesion molecules on EC. Pretreatment of EC with SCFA decreased adhesion of EHV-1 infected immune cells to EC and viral transfer (Poelaert et al., 2019a).

Cell-to-cell contacts activate the transcription and translation of viral proteins in infected CD172a^+^ cells and facilitate the transfer of new progeny virions to EC (Laval et al., 2015b). Infected T lymphocytes can also transfer virions to EC (Poelaert et al., 2019b). Using an *in vitro* flow system that mimics the rolling of PBMC to EC, Spiesschaert et al. (2015) demonstrated that EHV-1 gB and US3 proteins mediate viral transfer to EC in the presence of neutralizing antibodies. As a common strategy for herpesviruses, EHV-1 uses cell-cell (micro) fusion mechanisms for viral transmission (Laval et al., 2015b; Kamel et al., 2020). However, this transfer seems to be mainly non-productive (Laval et al., 2015b). These findings suggest that not all infected leukocytes transfer EHV-1 to EC, not every transfer leads to effective spread within the endothelium and not every successful infection of EC causes infection of surrounding tissues. Furthermore, it was shown that EHV-1 infection inhibits the type I IFN response in EC *in vitro* (Sarkar et al., 2015). After an early induction of type I IFN, EHV-1 suppresses the production of IFN while increasing the expression of late viral genes in EC. EHV-1 infection disrupts the interferon regulatory factor-3 (IRF-3) signaling pathway in EC through downregulation of endogenous IRF-3 protein level and inhibition of IRF3 nuclear translocation (Sarkar et al., 2016a). Additional studies showed that EHV-1 infection of EC suppresses the expression of TLR3 and TLR4 mRNA as well as the transcription of IRF7 and IRF9 mRNA in order to evade type I IFN antiviral effects. The virus also disrupts the JAK/STAT signaling pathway by degrading cellular levels of TYK2 and thus, downregulates downstream STAT1 and STAT2 phosphorylation levels (Sarkar et al., 2016b; Oladunni et al., 2019). The suppression of key factors in the induction of type I IFN only occurs after viral DNA synthesis, suggesting that late viral proteins are involved in the inhibition of STAT phosphorylation. The imbalance between antiviral and pro-inflammatory immune responses has been shown to play an important role in the clinical outcome of alphaherpesviruses infection *in vivo* (Laval et al., 2019). It is likely that EHV-1-mediated inhibition of type I IFN responses in EC directly promotes efficient viral replication and spread in the endothelium. In turn, this event favors the induction of a pro-inflammatory environment that contributes to the development of EHV-1-induced disease.

## Therapeutic Approaches Based on Current Knowledge of the Pathogenesis of EHV-1

Despite many years of research, there are currently no fully protective vaccines against EHV-1 and no efficient antiviral drugs. Current inactivated and live-attenuated vaccines fail to fully prevent virus shedding, cell-associated viremia and EHV-1-induced abortion and neurological disorders. The virus is still able to spread via a cell-associated viremia in vaccinated horses. Over the last 5 years, knowledge on the mechanisms of EHV-1 pathogenesis has dramatically increased. We can now think about designing new therapeutics that selectively target key immune evasive strategies developed by the virus at the level of the URT, blood circulation, and endothelium of target organs to prevent or mitigate future EHV-1 infections.

The first approach would be to prevent or restrict more efficiently primary EHV-1 infection. The integrity of the respiratory epithelium is often compromised by respiratory hazards (e.g., pollens, mycotoxins, mucolytic agents) present in the horse’s environment. The disruption of respiratory ICJ facilitates EHV-1 infection of the URT. The use of aerosol drugs that strengthen the respiratory epithelium may be an interesting strategy to modulate primary EHV-1 infection. These drugs should contain calcium to allow the stabilization of ICJ as well as protease inhibitors to act on foreign incoming proteases arising from pollens and other putative sources. The efficacy of aerosol therapy in the prophylaxis and treatment of EHV-1 disease is however speculative at this point (Van Cleemput et al., 2019a). Moreover, EHV-1 infection of respiratory epithelial cells induces the production of type I IFN that limits further spread of the virus within the epithelium. Still, the IFN response is not enough to prevent virus to migrate through the epithelium and infect immune cells. The oral use of recombinant type I IFN could be considered as an antiviral drug in EHV-1-infected horses to treat primary infection. So far, the low dose administration of human type I IFN to experimentally EHV-1 infected horses did not show significant decrease in viral shedding and clinical disease (Seahorn et al., 1990). The use of an equine recombinant type I IFN at a higher dose might be more appropriate for *in vivo* studies. Ultimately, the identification and subsequent targeting of EHV-1 basolateral receptor in the respiratory epithelium might be the best approach to fully block viral infection of the URT.

The second approach would be to halt the migration of immune cells to the primary site of infection and/or their subsequent infection. EHV-1-infected epithelial cells produce pro-inflammatory cytokines, chemokines, eBD2, and 3 to attract immune cells. The use of anti-inflammatory aerosol drugs that reduce the cytokine and chemokine responses (e.g., corticosteroids, cytokine/chemokine neutralizing antibodies, cytokine signaling blockers) could be considered in combination with antiherpesvirus therapies. As the mechanism of EHV-1 entry into target CD172a^+^ cells has been partially elucidated, it would be relevant to examine the efficacy of entry blockers as antivirals to prevent cell-associated viremia. Sialic acid residues present on CD172a^+^ cells are essential in the initiation of EHV-1 infection. The use of carbohydrate-binding molecules specific for sialic acid demonstrated protective *in vivo* efficacy against influenza virus and therefore could provide similar protection against EHV-1 infection and other pathogens that recognize sialic acid receptors (Connaris et al., 2014). As the replication of EHV-1 non-neurovirulent strain is delayed and controlled by HDAC in CD172a^+^ cells, it could be interesting to use specific HDAC inhibitors in combination with potent antiviral drugs to prevent cell-associated viremia. Highly selective HDAC inhibitors may relieve EHV-1 temporary block in viral gene expression and accelerate viral replication in monocytic cells. Subsequently, antiviral treatment may efficiently inhibit viral replication in these cells before they reach the target epithelium. Vaccination prior to HDAC inhibitors treatment could boost the immune response and also facilitate the clearance of infected cells. However, these approaches are contingent on the availability of potent antivirals and vaccine candidates on the market. As HDAC do not control replication of EHV-1 neurovirulent strains in CD172a^+^ cells, it is important to keep in mind that HDAC inhibitors may not be useful for horses with EHV-1-related neurological disorders or already showing symptoms.

The third approach would be to prevent the adhesion of EHV-1-infected immune cells to the endothelium of target organs. Transient administration of anti-adhesive agents that interfere with immune cell adhesion should be explored *in vivo* as a potential mitigation strategy for EHV-1-induced abortion and neurological disorders. A complete characterization of the adhesion molecules present on both immune cells and EC is beforehand required. Anti-inflammatory drugs have also been shown to decrease EC infection by reducing contact between EHV-1-infected PBMC and EC (Goehring et al., 2017). The administration of corticosteroids and anti-inflammatory drugs has been recommended for a short duration in EHM treatment, but no data has demonstrated their potential efficacy so far. SCFA have been shown to reduce leukocyte adhesion to EC *in vitro*. SCFA have anti-inflammatory properties and hence a high-fiber diet or diet supplemented with SFCA could potentially reduce systemic inflammation and ultimately prevent EHV-1-associated disease.

## Concluding Remarks

EHV-1 is able to impair horses’ health and affect equine industry all over the world. Over the last decade, the interest in EHV-1 has significantly increased due to frequent outbreaks of neurological disease and abortions caused by the virus. Within a global horse population of 58 million including 6 million in Europe, the high incidence of EHV-1 and severe associated symptoms raise serious health and economical concerns to the equine industry. Despite many years of research, no fully protective vaccines against EHV-1 or efficient antiviral drugs have been developed. Treatment is mainly limited to supportive therapy. The main reason is that EHV-1 uses a plethora of immune mechanisms to bypass the host’s immune response. A major breakthrough in our understanding of the pathogenesis of EHV-1 is therefore urgently needed. In this review, we summarize recent findings on the key immune evasive strategies used by the virus at main sites of infection: the URT, blood circulation, and endothelium of target organs. Their targeting may be a new and more effective therapeutic option to fight against EHV-1 infection.

## Author Contributions

KL suggested the idea and wrote the review. HN critically reviewed and corrected the review. All other authors read and approved the final manuscript. All authors contributed to the article and approved the submitted version.

## Conflict of Interest

KM was employed by company PERSEUS bvba. The remaining authors declare that the research was conducted in the absence of any commercial or financial relationships that could be construed as a potential conflict of interest.
